# Changes in Hippocampal Volume after Traumatic Brain Injury (TBI)

**DOI:** 10.21203/rs.3.rs-5390622/v1

**Published:** 2024-12-04

**Authors:** Kseniia Kriukova, Misque Boswell, Tuba Asifriyaz, Joyce Gong, Dominique Duncan, Paul Vespa

**Affiliations:** University of Southern California; University of Southern California; University of Southern California; University of Southern California; University of Southern California; Ronald Reagan UCLA Medical Center

**Keywords:** Traumatic Brain Injury (TBI), Hippocampal Volume, Post-Traumatic Epilepsy (PTE), Alzheimer’s disease (AD)

## Abstract

**Objective:**

To investigate hippocampal volume changes in moderate to severe traumatic brain injury (TBI) patients compared to healthy controls and assess their association with post-traumatic epilepsy (PTE), focusing on age-related effects.

**Methods:**

Imaging and demographic data for TBI patients were obtained from the Epilepsy Bioinformatics Study for Antiepileptogenic Therapy (EpiBioS4Rx) database; healthy controls matched by age and sex were sourced from Alzheimer’s Disease Neuroimaging Initiative (ADNI), the National Institute of Mental Health (NIMH) Intramural Healthy Volunteer Dataset, the Parkinson’s Progression Markers Initiative (PPMI), and the Autism Brain Imaging Data Exchange (ABIDE). MRI images for TBI subjects were obtained within 14–32 days post-injury. MRI data were preprocessed and segmented using FreeSurfer’s recon-all pipeline and the hippocampal subfield segmentation module.

**Results:**

TBI patients showed significantly smaller average hippocampal volumes than controls (β = −191.0, p = 8.33e−04, FDR p = 1.47e−03, Cohen’s d = −0.36), notably in the right and left CA1 head regions. No significant hippocampal volume differences were found between TBI+ and TBI− patients overall. In patients aged 60+, TBI+ patients had significantly larger volumes in the right CA1 head than TBI− patients (β = 57.43, p = 0.0300, FDR p = 0.040; Cohen’s d = 1.06).

**Conclusion:**

Hippocampal atrophy is evident in TBI patients but not directly linked to PTE development in most age groups. In patients over 60, hypertrophy in the right CA1 head may be associated with PTE, suggesting age and regional hippocampal changes influence epilepsy risk post-TBI. These findings highlight the complexity of hippocampal involvement in PTE and suggest potential shared mechanisms with neurodegenerative disorders such as Alzheimer’s disease.

## Introduction

### Posttraumatic Epilepsy

Posttraumatic epilepsy (PTE) is a severe complication of traumatic brain injury (TBI), affecting approximately 25 to 32% of patients with severe TBI (Pease et al. 2022). PTE increases the risk of disability and mortality and negatively impacts the quality of life for those affected (Karlander et al. 2022).

The definition of PTE has evolved. Jennet et al. initially defined it as recurrent unprovoked seizures resulting from TBI that manifest more than a week after the initial injury (Jennett 1969). Seizures occurring more than seven days after the injury are classified as late seizures (Jennett and van de Sande 1975). More recently, the International League Against Epilepsy (ILAE) broadened the criteria for epilepsy diagnosis to include 1) two seizures occurring more than 24 hours apart, 2) a single seizure with a recurrence risk exceeding 60% over a decade, or 3) diagnosis of an epilepsy syndrome. Under ILAE’s definition, in cases of TBI, a single late posttraumatic seizure (PTS) is sufficient to classify it as PTE due to the elevated likelihood of subsequent seizures (Fisher et al. 2014).

For the purposes of this study, we use the terms “late seizure” and “PTE” interchangeably, as a single late unprovoked seizure in individuals with severe TBI meets the ILAE criteria for an epilepsy diagnosis.

### Hippocampus in TBI

Hippocampal atrophy is a well-documented consequence of TBI, a finding supported by animal (Kotapka et al. 1991; Hicks et al. 1993) and human studies (Bigler et al. 1997; Serra-Grabulosa et al. 2005; Ariza et al. 2006; Cole et al. 2018; Harris et al. 2019).

The hippocampal head appears more prone to damage from TBI (Kotapka et al. 1991; Maxwell et al. 2003; Ariza et al. 2006). However, research is limited regarding how different parts of the hippocampus are affected by TBI. One speculated reason for the hippocampal head’s vulnerability is its high concentration of CA1 neurons (Ariza et al. 2006), which are important for memory and are linked to the frontobasal brain regions, making them particularly sensitive to injury (Gennarelli and Graham 1998). This sensitivity has also been demonstrated in cases of transient forebrain ischemia (Kotapka et al. 1991; Maxwell et al. 2003; Ariza et al. 2006).

Recent experimental work has provided further insights into hippocampal damage following TBI. Frankowski, Kim, and Hunt (2019) used a controlled cortical impact (CCI) injury model in CD1 mice to investigate hippocampal GABAergic interneuronal loss. At 30 days post-injury, they observed significant reductions in interneuron density. Specifically, approximately a 40% reduction throughout the dorsal hippocampus in the dentate gyrus and approximately a 36% reduction in the CA1 region. These findings underscore the vulnerability of these hippocampal areas to TBI-induced damage.

Additional evidence shows that hippocampal deterioration following TBI can be attributed to several critical molecular changes. Significant alterations in protein expression were observed within hippocampal subregions, particularly those involved in synaptic signaling, cytoskeletal organization, and energy metabolism (Maity et al. 2024). These changes disrupt normal cellular functions and contribute to neuronal damage. The study also noted upregulation of inflammation-related proteins, increased levels of proteins associated with oxidative stress, and alterations in proteins linked to neurodegenerative processes, such as tau pathology and amyloid precursor protein. Therefore, it is likely that these molecular alterations collectively contribute to the progressive deterioration of the hippocampus following trauma. Furthermore, a study on TBI-induced rats (Golarai et al. 2001) showed early changes post-TBI (1 day to 1 week), including significant cortical cell damage at impact site edges, which increased over time, also noting gross cell loss in the CA3 region of the hippocampus by 2–3 weeks after the injury. Collectively, these findings demonstrate the progression of hippocampal damage and its functional consequences following TBI.

### PTE Biomarkers

Hippocampal changes following TBI are significant and measurable, making them promising potential biomarkers for PTE. Previously, it was demonstrated that MRI-derived hippocampal position and orientation parameters effectively predict PTE, with notable changes in both ipsilateral and contralateral hippocampi, as shown in a rat lateral fluid percussion injury (FPI) model (De Feo et al. 2022). Other studies have identified additional hippocampal biomarkers, including reduced cerebral blood flow, associated with increased seizure susceptibility (Hayward et al. 2010) and early abnormalities in hippocampal morphology, which predicted later epilepsy development (Pitkänen and Engel 2014). Further distinct morphological changes in epileptic and non-epileptic rats post-injury were reported, developing a predictive model using PET parameters from the ipsilateral hippocampus (Shultz et al. 2013). These findings support the hippocampus as a sensitive indicator of PTE susceptibility, highlighting its potential for early detection and intervention in post-TBI epilepsy development.

### Other findings and gaps in research

Despite substantial research efforts, there remains a notable lack of validated biomarkers for predicting PTE in humans. Identifying reliable biomarkers for PTE/epileptogenesis is particularly challenging due to its multifaceted nature and the involvement of diverse biological processes (Duncan et al. 2018). Many biomarkers show significant associations with late seizures, but their variability remains high, likely due to heterogeneous study designs, inconsistent biomarker definitions, and diverse patient populations. A primary challenge is the underpowered nature of both animal and clinical studies, coupled with the lack of standardized methods, making comparative and translational analyses difficult.

Reliable biomarkers for PTE could significantly enhance research and treatment by identifying patients at high risk of developing epilepsy following TBI, enabling timely and targeted interventions (Garner et al. 2019). These biomarkers would help assess treatment the effectiveness of treatments without waiting for seizures to occur, monitor the progression of epileptogenesis, and guide the development of new therapies (Engel 2019).

Extensive research on chronic animal models of epilepsy, particularly those simulating hippocampal sclerosis, has enhanced our understanding of epileptogenesis. Given that hippocampal sclerosis is a common structural abnormality in mesial temporal lobe epilepsy, studying changes in hippocampal volume could be particularly informative for biomarker discovery (Pitkänen and Engel 2014).

Manninen et al. (2022) demonstrated that while hippocampal volume changes were statistically significant between sham animals and those with induced TBI, there was no significant difference in hippocampal volume between animals that developed posttraumatic epilepsy and those that did not.

This study aims to validate and extend previous findings on posttraumatic hippocampal atrophy in humans. We integrate neuroimaging and clinical data to investigate the relationship between hippocampal volume changes and epileptogenesis following TBI.

## Methods

### Subjects

The imaging and demographic data for TBI subjects were obtained from the Epilepsy Bioinformatics Study for Antiepileptogenic Therapy (EpiBioS4Rx) database (Vespa et al. 2019), housed on the Data Archive for the BRAIN Initiative (DABI) (Duncan et al. 2023; Subash et al. 2023). Data for matched healthy controls were sourced from the Alzheimer’s the National Institute of Mental Health (NIMH) Intramural Healthy Volunteer Dataset (Nugent et al. 2022), housed on the OpenNeuro (Markiewicz et al. 2021; Subash et al. 2023) archive, and from Disease Neuroimaging Initiative (ADNI) (Crawford et al. 2016), the Parkinson’s Progression Markers Initiative (PPMI) (Marek et al. 2011), and the Autism Brain Imaging Data Exchange (ABIDE) (Di Martino et al. 2014), all housed on Image and Data Archive (IDA) (Crawford et al. 2016).

Inclusion criteria for TBI subjects in the EpiBioS4Rx study were: 1) acute TBI patients with evidence of intracranial, 2) cortical and/or subcortical bleed on CT imaging; 3) age 6–100; 4) GCS 3–13 without continuous sedation at the time of enrollment; 5) ability to enroll within 72 hours of injury.

Exclusion criteria for TBI subjects were in the EpiBioS4Rx study: 1) diffuse axonal injury in the absence of hemorrhagic contusions or skull fracture; 2) isolated epidural hemorrhages that improve after evacuation; 3) no plan for continuous EEG monitoring during injury day 1–7; 4) inability to undergo MRI within post injury day 1–18 due to bullet, metal implant, pacemaker, etc.; 4) known HIV/AIDS, hepatitis B or C; pregnancy; 5) pre-existing neurologic disease (e.g. TBI, stroke, or neurodegenerative disorder); 6) pre-existing CNS malignancy; 7) pre-existing epilepsy/seizure disorder; 8) pre-existing dementia; 9) isolated anoxic brain injury; 10) devastating cervical spine injury; 11) brain death; 12) incarceration present or pending; 13) inability on part of patient or family to complete the study to Day 730; 14) positive SARS-CoV-2 (COVID-19) test; 15) any other condition or situation that the investigator believes may interfere with the safety of the subject or the conduct of the study, to include inability for study completion. For the current study, we also excluded subjects whose injury areas involved deep brain structures. To ensure the reliability of segmentation outcomes, we Specifically excluded subjects with injuries impacting deep brain structures, including the hippocampus. Trauma in these areas often compromises the quality of automatic segmentation, frequently leading to its failure.

The dataset used in this study includes 165 TBI patients: 101 subjects either completed a two-year follow-up without developing seizures or developed late PTS at least a week after the TBI (in cases where late PTS developed, completing the two-year follow-up was not considered necessary, as it was already confirmed that the patients had developed late PTS) and 65 with unknown seizure status. Among the 101 patients with known outcomes, 43 (42.6%) developed late seizures, while 58 (57.4%) did not. No statistically significant correlations were found between age, sex, GCS, and the occurrence of seizures. For the EpiBioS4Rx cohort, all images were obtained within 14 to 32 days after the trauma. Tables 1 and 2 summarize the age, sex, and GCS distribution for all TBI patients.

The healthy controls dataset used in this study includes 139 healthy control subjects. Table 3 provides a summary of the age and sex for healthy controls.

Healthy controls were matched to TBI subjects by age and sex, as both factors are known to influence hippocampal volume (Anstey et al. 2005; Wang et al. 2019).

Subjects were categorized into three groups: TBI who developed late seizures (TBI+), those with TBI who did not develop late seizures (TBI−), and healthy controls.

Based on the significance of the previous studies, we analyzed the volumes of the CA1 and CA3 regions and the total hippocampus. To reduce the bias caused by the right/left asymmetry of the hippocampus, we used the average of the right and left hippocampal volumes in healthy controls for comparison with TBI subjects.

For this study, T1-weighted images from 1.5T and 3T scanners were used. Due to the heterogeneity of the EpiBioS4Rx data, which includes clinically obtained images, we could not match the healthy controls’ images in terms of field strength, slice thickness, or scanning sequence. To address these discrepancies across healthy control subjects and within EpiBioS4Rx cohorts, we utilized the NeuroComBat package (R package ‘neuroCombat’) for correction (Fortin et al. 2017, 2018).

**Figure 1:**
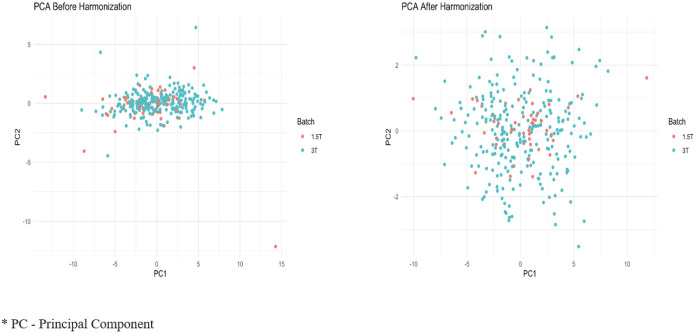
Comparative PCA Analysis of Hippocampal Volumes Before and After NeuroCombat Harmonization Across MRI Field Strengths

NeuroComBat harmonization has been shown to mitigate bias related to data heterogeneity, leading to consistent findings across various subjects and image measurements. In our analysis, the batch variable (field strength) and the model matrix were provided as inputs to the NeuroComBat function, which corrected for differences in data distributions across MRI field strengths. Figure 1 illustrates the principal component analysis (PCA) before and after harmonization. We acknowledge that data heterogeneity remains a limitation of this study (fig. 1).

### Preprocessing and Feature Extraction

**Figure 2:**
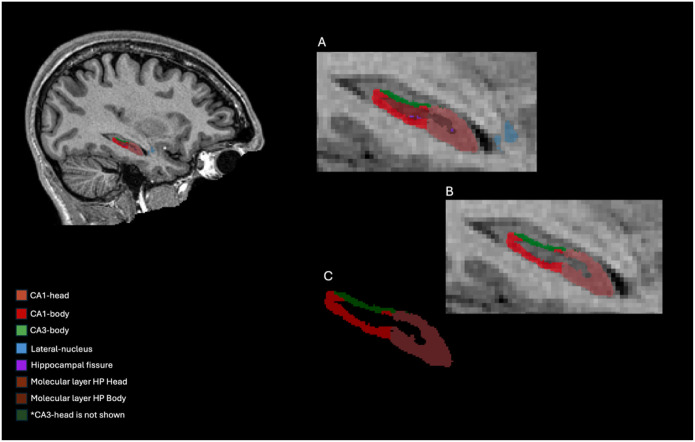
FreeSurfer Hippocampal segmentation. A. Sagittal view segmentation of the whole hippocampus with subject’s MRI for reference B. Sagittal view segmentation of relevant regions (CA1 head/body and CA3 head/body) with subject’s MRI for reference C. Sagittal view segmentation of relevant regions (CA1 head/body and CA3 head/body) without the reference image

Data were preprocessed and segmented using FreeSurfer’s recon-all (Fischl 2012) pipeline and the hippocampal subfield segmentation module (Iglesias et al. 2015). Recon-all includes 31 processing stages, including motion correction, intensity normalization, skull stripping, linear and non-linear volumetric registrations, white matter segmentation, spherical mapping and registration, and cortical mapping and registration (https://surfer.nmr.mgh.harvard.edu/fswiki/recon-all). The automated hippocampal subfield segmentation utilizes a probabilistic atlas built from ultra-high-resolution images and applied to subjects’ T1-weighted images (Iglesias et al. 2015). A sample of the FreeSurfer hippocampal segmentation is presented in Figure 2 (fig. 2).

All images were quality-checked before processing, and final segmentations were examined by a neurologist and a neuroradiologist. T2-weighted images were used for additional validation when necessary. Out of 192 initial images of TBI subjects, 20 failed FreeSurfer segmentation, and an additional 7 were excluded during the quality assessment. One subject was excluded due to preexisting cognitive problems. Of 144 segmented images of the healthy controls 139 passed the quality assessment. Healthy control data were assessed for outliers to minimize bias; none were excluded for reasons other than the segmentation quality.

### Analysis

The Shapiro-Wilk test was used to assess the normality of continuous variables to choose between parametric and non-parametric tests. Based on these results, the Wilcoxon rank-sum test was performed for the variables with non-normal distribution. Multiple linear regression analysis was used to assess differences in hippocampal volumes between TBI patients and healthy controls, adjusting for confounding variables such as age and sex. To account for multiple comparisons, statistical significance was determined using False Discovery Rate (FDR)-corrected p-values. Cohen’s d was calculated to quantify the effect size. All statistical analyses were conducted using R Version 2024.04.2+764.

## Results

### Healthy controls vs all TBI patients

**Figure 3:**
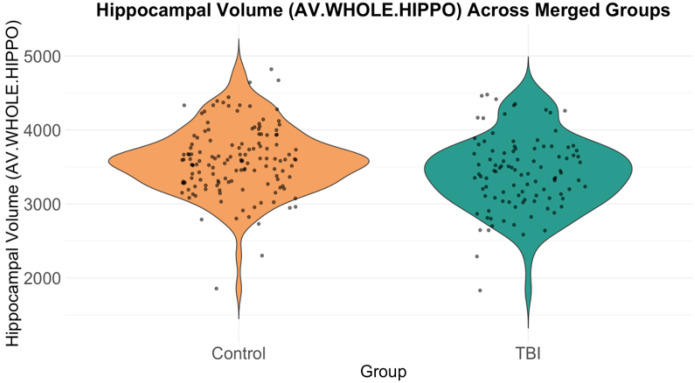
Violin plot showing the distribution of average whole hippocampal volumes for the control group and the TBI group

Multiple linear regression analysis, adjusted for age and sex, showed that TBI patients had significantly smaller average hippocampal volumes compared to healthy controls (β = −191.0, p = 8.33e-04, FDR-corrected p = 1.47e-03, Cohen’s d = −0.36) (fig. 3). Additionally, a significant negative association was found between age and hippocampal volume (β = −5.30, p = 1.58e-04, FDR-corrected p = 3.48e-04), while females exhibited significantly smaller hippocampal volumes compared to males (β = −376.0, p = 5.22e-08, FDR-corrected p = 1.77e-07).

**Figure 4:**
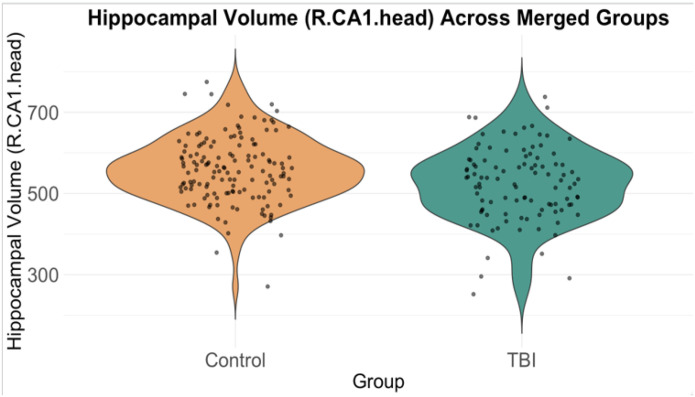
Violin plot showing the distribution of hippocampal volumes in the right CA1 head region for the control group and the TBI group

**Figure 5:**
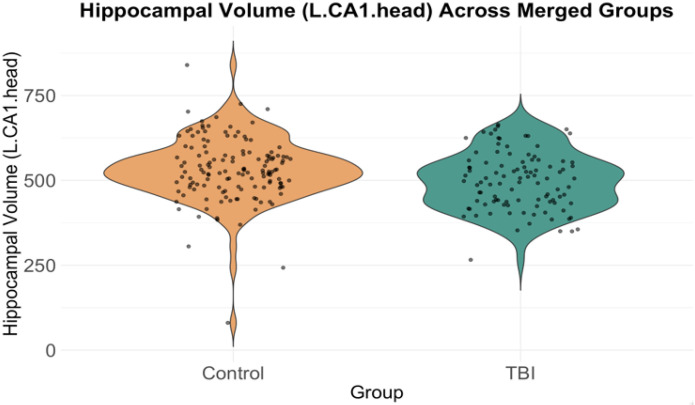
Violin plot showing the distribution of hippocampal volumes in the left CA1 head region for the control group and the TBI group

In the right CA1 head, TBI patients had significantly smaller volumes compared to healthy controls (β = −39.1, p = 2.49e-04, FDR-corrected p = 5.23e-04, Cohen’s d = −0.43) (fig. 4). Similarly, TBI patients showed significantly smaller volumes in the left CA1 head compared to healthy controls (β = −39.1, p = 2.49e-04, FDR-corrected p = 5.23e-04, Cohen’s d = −0.37) (fig. 5).

### TBI− vs TBI+

Multiple linear regression analysis, adjusted for age, sex, and emergency department arrival GCS scores, did not reveal significant differences in hippocampal volume across various subregions between TBI patients with and without seizures. While TBI patients were matched to controls by age in the earlier analysis, this matching was not applied within the TBI group (i.e. between TBI patients with and without seizures). Given the considerable age variability in the TBI group and the known influence of age on post-TBI recovery and seizure development, a subgroup analysis based on age to account for these potential age-related effects was performed.

**Figure 6:**
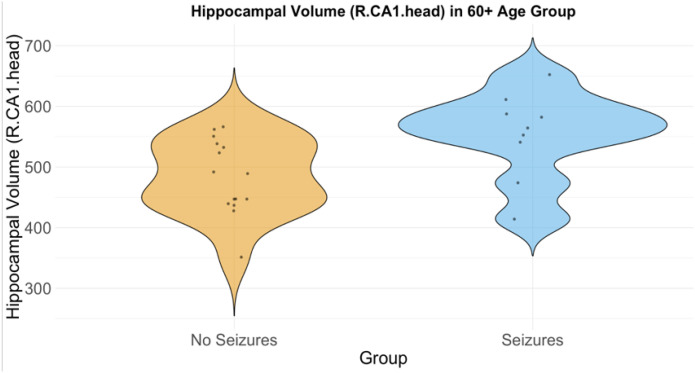
Violin plot showing the distribution hippocampal volumes in the right CA1 head region for patients with and without Seizures (60+ Age Group)

Multiple linear regression analysis, adjusted for sex and GCS, showed that seizure development was significantly associated with larger hippocampal volumes in the right CA1 head region for the 60+ age group (β = 57.43, p = 0.0300, FDR-corrected p = 0.040; Cohen’s d = 1.06) (fig.6). While no significant association was found between GCS at arrival and hippocampal volume (β = −1.08, p = 0.754, FDR-corrected p = 0.769), females exhibited significantly smaller hippocampal volumes compared to males in this region (β = −95.00, p = 0.0034, FDR-corrected p = 0.0067).

No statistically significant results were found for the other hippocampal regions or age groups after applying FDR correction.

## Discussion

Hippocampal atrophy following TBI has been observed in humans, consistent with findings from animal models (Hicks et al. 1993). An early study by Bigler et al. (1997) examined hippocampal volume changes in TBI patients aged 16 to 65. They found a 9% reduction in hippocampal volume in early TBI patients (within 100 days post-injury), while late-stage TBI patients (beyond 100 days) showed a more significant reduction than the control group.

In a notable study, Ariza et al. (2006) investigated hippocampal volumes in 20 moderate to severe TBI patients, ages 17 to 39, matched with healthy controls. They found that TBI patients showed hippocampal atrophy, with the hippocampal head particularly affected.

Further, Jorge et al. (2007) reported that patients with severe TBI, ages 16 to 65, had significantly smaller hippocampi compared to those with mild TBI.

Several animal studies have shown that TBI-induced cell loss in the hippocampus may increase seizure susceptibility, similar to hippocampal sclerosis (Lowenstein et al. 1992; Cavazos et al. 1994; Toth et al. 1998; Tate and Bigler 2000; Golarai et al. 2001). This increased risk is attributed to the loss of inhibitory neurons, leading to an imbalance favoring excitation, potentially contributing to the elevated risk of seizure disorders, including PTE (Golarai et al. 2001).

Swarts et al. (2006) compared hippocampal pathological changes in subjects with temporal lobe epilepsy (TLE) and TBI, finding similar patterns. They identified a connection between these changes and PTE occurrence, but their paper lacked a detailed analysis of this link, leaving the implications somewhat unclear. It remains uncertain whether these findings are common in all TBI patients or occur more frequently in those who later develop epilepsy.

Vespa et al. (2010) demonstrated that early electrographic seizures are associated with long-term selective atrophy of the ipsilateral hippocampus and, to a lesser extent, the contralateral hippocampus. This suggests a localized impact without affecting overall brain atrophy. However, their analysis did not cover late seizures.

To our knowledge, no studies have Specifically investigated hippocampal volume changes in moderate to severe TBI patients concerning PTE occurrence. Our study, which included a significant number of participants over 60, differs from previous research that focused primarily on individuals aged 16 to 65. While previous studies indicate hippocampal changes occur after brain trauma and may be linked to PTE, it remains unclear whether these changes directly increase the likelihood of late seizures.

Our analysis of hippocampal volumes in TBI patients versus controls is consistent with prior research (Hicks et al. 1993; Bigler et al. 1997; Ariza et al. 2006; Swartz et al. 2006). Additionally, our findings corroborate Ariza et al.’s (2006) observations about the CA1 head region’s vulnerability to cell loss.

Interestingly, when comparing hippocampal volumes of TBI patients with and without post-traumatic epilepsy (TBI+ vs. TBI−), we found no significant differences across most age groups. However, in the 60+ age group, the CA1 head region was larger in TBI+ patients compared to TBI− patients, challenging previous assumptions.

These findings highlight two key points: 1) Hippocampal volume loss occurs after trauma but is not necessarily linked to PTE, aligning with previous findings (Manninen et al. 2022), and 2) CA1 head hypertrophy may be associated with PTE development in patients over 60.

This suggests that while hippocampal damage commonly follows TBI, its impact on epilepsy development may vary based on specific regional changes within the hippocampus and possibly age.

Our findings that CA1 head hypertrophy may be associated with PTE development in patients over 60 led us to consider the growing interest in exploring potential commonalities between Alzheimer’s disease (AD) and epileptogenesis (Fang et al. 2023).

Notably, AD patients have an increased risk for epileptic seizures (Vöglein et al. 2020), tend to exhibit neuronal hypertrophy, particularly in the hippocampal CA1 region (Iacono et al. 2008). This correlation suggests that similar neural adaptation mechanisms might be at play in both conditions, potentially offering insights into their interconnections and underlying pathophysiology.

In a 2008 study on the Baltimore Longitudinal Study of Aging (BLSA) (Kawas et al. 2000), autopsy samples showed CA1 hypertrophy in asymptomatic Alzheimer’s disease (ASYMAD) subjects between 71 and 96 years old (Iacono et al. 2008). Subjects had increased volumes of neuronal bodies, nuclei, and nucleoli in the CA1 of the hippocampus region only when compared with controls and mild cognitive impairment (MCI) samples. Interestingly, cognitively impaired patients with Alzheimer’s pathology showed significant atrophy in the same region. These observations initially seemed to point to compensatory mechanisms that would have been at play, preventing patients from showing the symptoms of AD. However, it has been shown that neuronal hyperactivation occurs in early AD (Targa Dias Anastacio et al. 2022), which has been associated with neurogenic activity, while later stages of the disease show neuronal hypoactivation (Dickerson et al. 2005; Celone et al. 2006), consistent with neuronal loss. Therefore, neuronal hyperactivity may be predictive of subsequent hypoactivity and decline. Further confirming the pathogenic effects of hyperexcitability in MCI patients, antiepileptogenic drugs have shown reduced hyperexcitability, followed by improved memory performance (Bakker et al. 2012; Sanchez et al. 2012).

AD patients are at an increased risk of developing seizures and epilepsy, and changes in the CA1 are visible in both disorders (Friedman et al. 2012); thus, the explanation for CA1 enlargement early in the disease could be a shared mechanism for subsequent deterioration.

## Figures and Tables

**Table 1.** Summary of the age, sex, and GCS distribution for TBI patients.

Outcome	Min Age	Max Age	Mean Age	SD Age	Min GCS	Max GCS	Mean GCS	SD GCS	Male (n)	Male (%)	Female (n)	Female (%)
All TBI	7	90	43.4	20.3	3	15	7.73	3.76	128	77.6	37	22.4
TBI−	7	90	44.2	20.9	3	15	8.34	3.90	45	44.6	13	12.9
TBI+	15	71	40.3	17.5	3	15	7.12	3.44	37	36.6	6	5.94

**Table 2.** Summary of the age TBI patients with and without seizures.

Age Group	Total subjects	TBI+	TBI−
0–18	10	4	6
18–40	38	19	19
40–60	29	11	18
60+	24	9	15

**Table 3.** Summary of the age and sex distribution for healthy control subjects.

Min Age	Max Age	Mean Age	SD Age	Male (n)	Male (%)	Female (n)	Female (%)
8	90	41.8	20.7	104	74.8	35	25.2

## Data Availability

*DABI* (EpiBios4Rx): doi.org/10.18120/hvkb-z638 *OpenNeuro* (IHVD): doi:10.18112/openneuro.ds004215.v2.0.1 *IDA* (ADNI): https://ida.loni.usc.edu/home/projectPage.jsp?project=ADNI *IDA* (ABIDE): https://ida.loni.usc.edu/home/projectPage.jsp?project=ABIDE *IDA* (PPMI): ): https://ida.loni.usc.edu/home/projectPage.jsp?project=PPMI

## References

[1] Anstey K, Maller J, Meslin C, (2005) Hippocampal and amygdalar volumes in relation to handedness in adults aged 60–64. Neuroreport 15:2825–9 15597062

[2] Ariza M, Serra-Grabulosa JM, Junqué C, (2006) Hippocampal head atrophy after traumatic brain injury. Neuropsychologia 44:1956–1961. 10.1016/j.neuropsychologia.2005.11.007 16352320

[3] Bakker A, Krauss GL, Albert MS, (2012) Reduction of Hippocampal Hyperactivity Improves Cognition in Amnestic Mild Cognitive Impairment. Neuron 74:467–474. 10.1016/j.neuron.2012.03.023 22578498 PMC3351697

[4] Bigler ED, Blatter DD, Anderson CV, (1997) Hippocampal Volume in Normal Aging and Traumatic Brain Injury PMC8337859 9010515

[5] Cavazos JE, Das I, Sutula TP (1994) Neuronal loss induced in limbic pathways by kindling: evidence for induction of hippocampal sclerosis by repeated brief seizures. J Neurosci 14:3106–3121. 10.1523/JNEUROSCI.14-05-03106.1994 8182460 PMC6577461

[6] Celone KA, Calhoun VD, Dickerson BC, (2006) Alterations in Memory Networks in Mild Cognitive Impairment and Alzheimer’s Disease: An Independent Component Analysis. J Neurosci 26:10222–10231. 10.1523/JNEUROSCI.2250-06.2006 17021177 PMC6674636

[7] Cole JH, Jolly A, De Simoni S, (2018) Spatial patterns of progressive brain volume loss after moderate-severe traumatic brain injury. Brain 141:822–836. 10.1093/brain/awx354 29309542 PMC5837530

[8] Crawford KL, Neu SC, Toga AW (2016) The Image and Data Archive at the Laboratory of Neuro Imaging. NeuroImage 124:1080–1083. 10.1016/j.neuroimage.2015.04.067 25982516 PMC4644502

[9] De Feo R, Manninen E, Chary K, (2022) Hippocampal position and orientation as prognostic biomarkers for posttraumatic epileptogenesis: An experimental study in a rat lateral fluid percussion model. Epilepsia 63:1849–1861. 10.1111/epi.17264 35451496 PMC9283326

[10] Di Martino A, Yan C-G, Li Q, (2014) The autism brain imaging data exchange: towards a large-scale evaluation of the intrinsic brain architecture in autism. Mol Psychiatry 19:659–667. 10.1038/mp.2013.78 23774715 PMC4162310

[11] Dickerson BC, Salat DH, Greve DN, (2005) Increased hippocampal activation in mild cognitive impairment compared to normal aging and AD. Neurology 65:404–411. 10.1212/01.wnl.0000171450.97464.49 16087905 PMC4335677

[12] Duncan D, Barisano G, Cabeen R, (2018) Analytic Tools for Post-traumatic Epileptogenesis Biomarker Search in Multimodal Dataset of an Animal Model and Human Patients. Front Neuroinform 12:86. 10.3389/fninf.2018.00086 30618695 PMC6307529

[13] Duncan D, Garner R, Brinkerhoff S, (2023) Data Archive for the BRAIN Initiative (DABI). Scientific Data 10:83 36759619 10.1038/s41597-023-01972-z PMC9911697

[14] Engel J (2019) Epileptogenesis, Traumatic Brain Injury, and Biomarkers. Neurobiol Dis 123:3–7. 10.1016/j.nbd.2018.04.002 29625256 PMC6170720

[15] Fang Y, Si X, Wang J, (2023) Alzheimer Disease and Epilepsy. Neurology 101:e399–e409. 10.1212/WNL.0000000000207423 37225432 PMC10435057

[16] Fischl B (2012) FreeSurfer. NeuroImage 62:774–781. 10.1016/j.neuroimage.2012.01.021 22248573 PMC3685476

[17] Fisher RS, Acevedo C, Arzimanoglou A, (2014) ILAE Official Report: A practical clinical definition of epilepsy. Epilepsia 55:475–482. 10.1111/epi.12550 24730690

[18] Fortin J-P, Cullen N, Sheline YI, (2018) Harmonization of cortical thickness measurements across scanners and sites. NeuroImage 167:104–120. 10.1016/j.neuroimage.2017.11.024 29155184 PMC5845848

[19] Fortin J-P, Parker D, Tunç B, (2017) Harmonization of multi-site diffusion tensor imaging data. NeuroImage 161:149–170. 10.1016/j.neuroimage.2017.08.047 28826946 PMC5736019

[20] Frankowski JC, Kim YJ, Hunt RF (2019) Selective vulnerability of hippocampal interneurons to graded traumatic brain injury. Neurobiology of Disease 129:208–216. 10.1016/j.nbd.2018.07.022 30031783 PMC6690377

[21] Friedman D, Honig LS, Scarmeas N (2012) Seizures and Epilepsy in Alzheimer’s Disease. CNS Neuroscience & Therapeutics 18:285–294. 10.1111/j.1755-5949.2011.00251.x 22070283 PMC3630499

[22] Garner R, La Rocca M, Vespa P, (2019) Imaging biomarkers of posttraumatic epileptogenesis. Epilepsia 60:2151–2162. 10.1111/epi.16357 31595501 PMC6842410

[23] Gennarelli TA, Graham DI (1998) Neuropathology of the Head Injuries. Semin Clin Neuropsychiatry 3:160–175 10085204

[24] Golarai G, Greenwood AC, Feeney DM, Connor JA (2001) Physiological and Structural Evidence for Hippocampal Involvement in Persistent Seizure Susceptibility after Traumatic Brain Injury. J Neurosci 21:8523–8537. 10.1523/JNEUROSCI.21-21-08523.2001 11606641 PMC6762822

[25] Harris TC, De Rooij R, Kuhl E (2019) The Shrinking Brain: Cerebral Atrophy Following Traumatic Brain Injury. Ann Biomed Eng 47:1941–1959. 10.1007/s10439-018-02148-2 30341741 PMC6757025

[26] Hayward NM, Immonen R, Tuunanen PI, (2010) Association of Chronic Vascular Changes with Functional Outcome after Traumatic Brain Injury in Rats. Journal of Neurotrauma 27:2203–2219. 10.1089/neu.2010.1448 20839948

[27] Hicks RR, Smith DH, Lowenstein DH, (1993) Mild Experimental Brain Injury in the Rat Induces Cognitive Deficits Associated with Regional Neuronal Loss in the Hippocampus. Journal of Neurotrauma 10:405–414. 10.1089/neu.1993.10.405 8145264

[28] Iacono D, O’Brien R, Resnick SM, (2008) Neuronal Hypertrophy in Asymptomatic Alzheimer Disease. Journal of Neuropathology & Experimental Neurology 67:578–589. 10.1097/NEN.0b013e3181772794 18520776 PMC2518071

[29] Iglesias JE, Augustinack JC, Nguyen K, (2015) A computational atlas of the hippocampal formation using *ex vivo*, ultra-high resolution MRI: Application to adaptive segmentation of *in vivo* MRI. NeuroImage 115:117–137. 10.1016/j.neuroimage.2015.04.042 25936807 PMC4461537

[30] Jennett B, van de Sande J (1975) EEG Prediction of Post-Traumatic Epilepsy. Epilepsia 16:251–256. 10.1111/j.1528-1157.1975.tb06055.x 807472

[31] Jennett WB (1969) EARLY TRAUMATIC EPILEPSY: Definition and Identity. The Lancet 293:1023–1025. 10.1016/S0140-6736(69)91822-4 4181257

[32] Jorge RE, Acion L, Starkstein SE, Magnotta V (2007) Hippocampal Volume and Mood Disorders After Traumatic Brain Injury. Biological Psychiatry 62:332–338. 10.1016/j.biopsych.2006.07.024 17123480

[33] Karlander M, Ljungqvist J, Sörbo A, Zelano J (2022) Risk and cause of death in post-traumatic epilepsy: a register-based retrospective cohort study. J Neurol 269:6014–6020. 10.1007/s00415-022-11279-5 35852600 PMC9553825

[34] Kawas C, Gray S, Brookmeyer R, (2000) Age-specific incidence rates of Alzheimer’s disease. Neurology 54:2072–2077. 10.1212/WNL.54.11.2072 10851365

[35] Kotapka MJ, Gennarelli TA, Graham DI, (1991) Selective Vulnerability of Hippocampal Neurons in Acceleration-Induced Experimental Head Injury. Journal of Neurotrauma 8:247–258. 10.1089/neu.1991.8.247 1803033

[36] Lowenstein DH, Thomas MJ, Smith DH, McIntosh TK (1992) Selective vulnerability of dentate hilar neurons following traumatic brain injury: a potential mechanistic link between head trauma and disorders of the hippocampus. J Neurosci 12:4846–4853. 10.1523/JNEUROSCI.12-12-04846.1992 1464770 PMC6575779

[37] Maity S, Huang Y, Kilgore MD, (2024) Mapping dynamic molecular changes in hippocampal subregions after traumatic brain injury through spatial proteomics. Clin Proteom 21:32. 10.1186/s12014-024-09485-6 PMC11089002 38735925

[38] Manninen E, Chary K, De Feo R, (2022) Acute Hippocampal Damage as a Prognostic Biomarker for Cognitive Decline but Not for Epileptogenesis after Experimental Traumatic Brain Injury. Biomedicines 10:2721. 10.3390/biomedicines10112721 36359242 PMC9687561

[39] Marek K, Jennings D, Lasch S, (2011) The Parkinson Progression Marker Initiative (PPMI). Prog Neurobiol 95:629–635. 10.1016/j.pneurobio.2011.09.005 21930184 PMC9014725

[40] Markiewicz CJ, Gorgolewski KJ, Feingold F, (2021) The OpenNeuro resource for sharing of neuroscience data. Elife 10:e71774 34658334 10.7554/eLife.71774 PMC8550750

[41] Maxwell WL, Dhillon K, Harper L, (2003) There Is Differential Loss of Pyramidal Cells from the Human Hippocampus with Survival after Blunt Head Injury. Journal of Neuropathology & Experimental Neurology 62:272–279. 10.1093/jnen/62.3.272 12638731

[42] Nugent AC, Thomas AG, Mahoney M, (2022) The NIMH intramural healthy volunteer dataset: A comprehensive MEG, MRI, and behavioral resource. Sci Data 9:518. 10.1038/s41597-022-01623-9 36008415 PMC9403972

[43] Pease M, Gonzalez-Martinez J, Puccio A, (2022) Risk Factors and Incidence of Epilepsy after Severe Traumatic Brain Injury. Ann Neurol 92:663–669. 10.1002/ana.26443 35713346 PMC9489614

[44] Pitkänen A, Engel J (2014) Past and Present Definitions of Epileptogenesis and Its Biomarkers. Neurotherapeutics 11:231–241. 10.1007/s13311-014-0257-2 24492975 PMC3996117

[45] Sanchez PE, Zhu L, Verret L, (2012) Levetiracetam suppresses neuronal network dysfunction and reverses synaptic and cognitive deficits in an Alzheimer’s disease model. Proceedings of the National Academy of Sciences 109:E2895–E2903. 10.1073/pnas.1121081109 PMC3479491 22869752

[46] Serra-Grabulosa JM, Junqué C, Verger K, (2005) Cerebral correlates of declarative memory dysfunctions in early traumatic brain injury. Journal of Neurology, Neurosurgery & Psychiatry 76:129–131. 10.1136/jnnp.2004.027631 15608014 PMC1739340

[47] Shultz SR, Cardamone L, Liu YR, (2013) Can structural or functional changes following traumatic brain injury in the rat predict the epileptic outcome? Epilepsia 54:1240–1250. 10.1111/epi.12223 23718645 PMC4032369

[48] Subash P, Gray A, Boswell M, (2023) A comparison of neuroelectrophysiology databases. Sci Data 10:719. 10.1038/s41597-023-02614-0 37857685 PMC10587056

[49] Swartz BE, Houser CR, Tomiyasu U, (2006) Hippocampal Cell Loss in Posttraumatic Human Epilepsy. Epilepsia 47:1373–1382. 10.1111/j.1528-1167.2006.00602.x 16922884

[50] Targa Dias Anastacio H, Matosin N, Ooi L (2022) Neuronal hyperexcitability in Alzheimer’s disease: what are the drivers behind this aberrant phenotype? Transl Psychiatry 12:1–14. 10.1038/s41398-022-02024-7 35732622 PMC9217953

[51] Tate DF, Bigler ED (2000) Fornix and Hippocampal Atrophy in Traumatic Brain Injury. Learn Mem 7:442–446. 10.1101/lm.33000 11112803

[52] Toth Z, Yan X-X, Haftoglou S, (1998) Seizure-Induced Neuronal Injury: Vulnerability to Febrile Seizures in an Immature Rat Model. J Neurosci 18:4285–4294. 10.1523/JNEUROSCI.18-11-04285.1998 9592105 PMC3387924

[53] Vespa PM, McArthur DL, Xu Y, (2010) Nonconvulsive seizures after traumatic brain injury are associated with hippocampal atrophy. Neurology 75:792–798. 10.1212/WNL.0b013e3181f07334 20805525 PMC2938965

[54] Vespa PM, Shrestha V, Abend N, (2019) The Epilepsy Bioinformatics Study for Anti-Epileptogenic Therapy (EpiBioS4Rx) Clinical Biomarker: Study Design and Protocol. Neurobiol Dis 123:110–114. 10.1016/j.nbd.2018.07.025 30048805 PMC6344322

[55] Vöglein J, Ricard I, Noachtar S, (2020) Seizures in Alzheimer’s disease are highly recurrent and associated with a poor disease course. J Neurol 267:2941–2948. 10.1007/s00415-020-09937-7 32488295 PMC7501095

[56] Wang Y, Xu Q, Luo J, (2019) Effects of Age and Sex on Subcortical Volumes. Front Aging Neurosci 11:. 10.3389/fnagi.2019.00259 PMC6775221 31616285

